# Hybrid 1D/3D-Structured Perovskite as a Highly Selective and Stable Sensor for NO_2_ Detection at Room Temperature

**DOI:** 10.3390/molecules28062615

**Published:** 2023-03-13

**Authors:** Anqi Cheng, Jinru Zhao, Xi-Ao Wang, Zhen Lu, Yan Qi, Jiankun Sun

**Affiliations:** College of Chemistry and Chemical Engineering, Qingdao University, Qingdao 266071, China

**Keywords:** gas sensor, NO_2_ detection, 1D/3D hybrid-structured perovskite, room temperature, stability

## Abstract

To exploit high-performance and stable sensing materials with a room working temperature is pivotal for portable and mobile sensor devices. However, the common sensors based on metal oxide semiconductors usually need a higher working temperature (usually above 300 °C) to achieve a good response toward gas detection. Currently, metal halide perovskites have begun to rise as a promising candidate for gas monitoring at room temperature but suffer phase instability. Herein, we construct 1D/3D PyPbI_3_/FA_0.83_Cs_0.17_PbI_3_ (denoted by PyPbI_3_/FACs) bilayer perovskite by post-processing spin-coating Pyrrolidinium hydroiodide (PyI) salt on top of 3D FACs film. Benefitting from the 1D PyPbI_3_ coating layer, the phase stability of 1D/3D PyPbI_3_/FACs significantly improves. Simultaneously, the gas sensor based on the 1D/3D PyPbI_3_/FACs bilayer perovskite presents a superior selectivity and sensitivity toward NO_2_ detection at room temperature, with a low detection limit of 220 ppb. Exposed to a 50 ± 3% relative humidity (RH) level environment for a consecutive six days, the 1D/3D PyPbI_3_/FACs perovskite-based sensor toward 10 ppm NO_2_ can still maintain a rapid response with a slight attenuation. Gas sensors based on hybrid 1D/3D-structured perovskite in this work may provide a new pathway for highly sensitive and stable gas sensors in room working temperature, accelerating its practical application and portable device.

## 1. Introduction

Environmental pollution has been a social issue and attracted extensive attention. Especially air contaminations, such as carbides, sulfides, nitrogen compounds and volatile organic compound (VOC) gases, can cause serious damage to the environment and human health [1,2,3,4]. Among them, nitrogen dioxide (NO_2_) is an extremely common contamination in air atmosphere, predominantly originating from the massive discharge of fossil-fuel combustion and motor vehicle exhaust, but NO_2_ with a ppm-level concentration can cause serious respiratory diseases, lung damage, neurasthenia and even endanger life safety [5,6,7]. Therefore, reliable and convenient detection of NO_2_ in the surrounding environment is closely relevant to humankind’s daily life. Currently, gas monitoring technology contains gas/liquid chromatography methods, acoustic sensors, chemical resistance sensors, electrochemical sensors and optical sensors, etc. [8]. In terms of equipment operation, cost, gas selectivity and response performance, chemical resistance sensors have been considered the most promising gas sensor. The chemical resistance sensors based on the metal oxide layer [9] have also been widely studied as NO_2_ gas sensors due to their stability and low cost. However, a high operating temperature of the common metal oxides-based sensor (usually above 300 °C) increases the power consumption and security issues, which impedes further commercial application in portable and mobile sensor devices. Therefore, exploiting efficient sensing materials for NO_2_ monitoring at room temperature is pivotal for practical application and is urgently desirable.

Organic-inorganic hybrid metal halide perovskites have recently attracted tremendous scientific research owing to their unique photoelectric properties and low-temperature solution processability, presenting a potential candidate in the field of solar photovoltaics, light-emitting diodes, laser, photodetectors, etc. [10,11,12,13]. More interestingly, in recent years, perovskites have begun to rise as a promising sensor material in detecting NO_2_, oxygen, ozone, ammonia, sulfur dioxide, humidity, organic vapors [9,14,15,16,17,18,19], etc. For instance, Fang et al. in 2016 demonstrated that methylammonium lead tribromide (MAPbBr_3_) single crystal showed an ultrahigh sensitivity toward the environmental atmosphere, including oxygen and water vapor [16]. Zhu et al. reported that CH_3_NH_3_PbBr_3_ perovskite nanocrystal film exhibited a good response to NO_2_ gas at room temperature [20]. Sensors based on FAPbCl_3_ are demonstrated as highly selective NH_3_ sensors with a detection threshold of 50 ppb [21]. Despite the inspiring development, three-dimensional (3D) perovskite still suffers from poor moisture and thermal and optical stability due to its intrinsic structural characteristics [22]. MAPbI_3_ has demonstrated inherent thermal instability [23]. By comparison, formamidinium (FA)-based FAPbI_3_ tends to have more research showing superior thermal stability, but FAPbI_3_ occurs a phase instability, which can spontaneously transform from desired black α-phase to unsatisfactory yellow δ-phase under ambient conditions [24,25,26], impeding its practical application and development.

To address the issue of FAPbI_3_ phase instability, various strategies, such as doping, interface engineering, additive engineering, etc., have been attempted [27,28,29,30,31,32,33,34,35,36]. Mixing proper cations and anions into perovskite structure, including MA^+^, Cs^+^, Rb^+^, Br^−^, etc., has been demonstrated to stabilize α-phase FAPbI_3_ [37,38,39,40], availing the power conversion efficiency and work lifetime of PSCs solar cells device [41], but reduced thermal stability occurs. Recently, low-dimensional perovskite functionalized 3D perovskite layers developed by introducing larger organic cations into 3D perovskite to passivate boundaries via one-step or post-processing to build heterojunction-structured perovskite has exhibited a significant improvement of FA-based black perovskite phase stability and device performance [22,42]. Because low-dimensional perovskite coating on the surface of 3D perovskite can play a protective-layer role, it can effectively prevent water molecules from invading the 3D perovskite polycrystalline film to prolong the perovskite’s operating life [42,43,44]. Especially, 1D perovskite possesses both thermal stability and water resistance due to its inherent structural characteristics, as 1D perovskite molecules, such as TAPbI_3_ (TAI: thiazole ammonium iodide), FEAPbI_3_ (FEA: 2,2,2-trifluoroethan-1-amine) and DMAPbI_3_ (DMAI: dimethylamine iodide) [44,45], have been demonstrated as an efficient way to improve the efficiency and stability of perovskite solar cells in the field of solar cells. Among them, PyI with five-membered hetero-cycles draws great attention due to a low ring strain, suitable tolerance factor of 1.026 [46] and intrinsic hydrophobicity. Several works also reported that PyI reacted with 3D MAPbI_3_ to form 1D/3D-hybrid perovskite, which leads to a substantial enhancement of environmental stability of solar cells device [47,48,49]. Xu et al. theoretically reveal that a 1D/3D bilayer structure constructed by 1D PyPbI_3_ can prompt the stability of FA-based perovskite in thermodynamics and kinetics [22]. However, the outstanding stability feature, the application research of 1D/3D perovskite, is still in its primary stage and mainly focuses on solar cells. Especially, 3D FA-based perovskite as a sensor material for gas detection has rarely been studied, much less a constructed 1D/3D bilayer perovskite with superior stability for low-concentrated NO_2_ detection at room temperature.

Herein, inspired by the former work, we constructed a 1D/3D hybrid-structured perovskite by post-treatment spinning Pyrrolidinium hydroiodide (PyI) salt on top of 3D FA_0.83_Cs_0.17_PbI_3_ (denoted by FACs) film and assembled it into a NO_2_ gas sensor. The 1D/3D bilayer perovskite-based sensor shows a superior gas response and selectivity toward NO_2_ at room temperature in the ambient environment. Moreover, benefitting from the 1D PyPbI_3_ coating layer, the 1D/3D PyPbI_3_/FACs bilayer perovskite-based sensor presents superior long-term stability, which can almost maintain the original phase and response without few attenuations when exposed to high humidity conditions for a successive six days. Our finding may open up a new application pathway of 1D/3D hybrid-structured perovskite, which accelerate the development of high-stability gas sensors as NO_2_ monitoring under room temperature and high humidity atmosphere.

## 2. Results and Discussion

### 2.1. Structure and Characterizations

**Figure 1:**
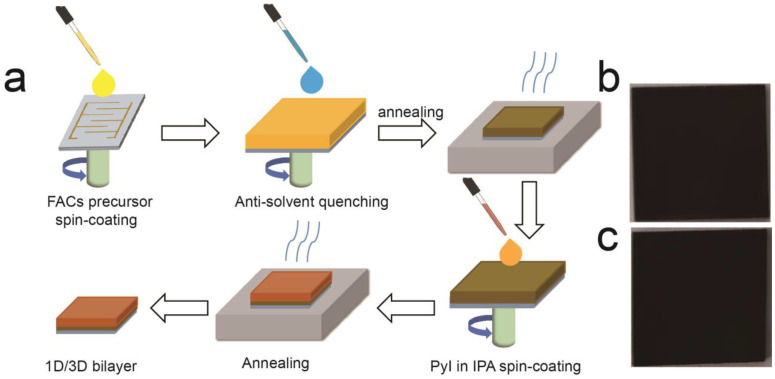
(**a**) Schematic illustration of the fabrication process of 1D/3D hybrid-structured perovskite-based sensor. Photographs of 3D FACs (**b**) and 1D/3D perovskite (**c**).

As illustrated in Figure 1a, the 1D/3D PyPbI_3-_and FACs perovskite-based sensor was prepared by the post-processing spin-coating method. Firstly, 3D FA_0.83_Cs_0.17_PbI_3_ (FACs) perovskite deposited on the gold interdigitated electrode was synthesized by ethyl acetate (EA) antisolvent quenching and annealing at 170 °C. Then, Pyrrolidinium hydroiodide (PyI) in isopropyl alcohol (IPA) solution was dropped on the spinning 3D FACs film. After spin-coating and annealing, a 1D/3D PyPbI_3_/FACs bilayer perovskite film was obtained. More detailed experiment procedures are in the Supplementary Materials. As the photographs show in Figure 1b and c, both 3D FACs and 1D/3D PyPbI_3_/FACs perovskite film display a homogeneous black and seem to have no obvious difference.

**Figure 2:**
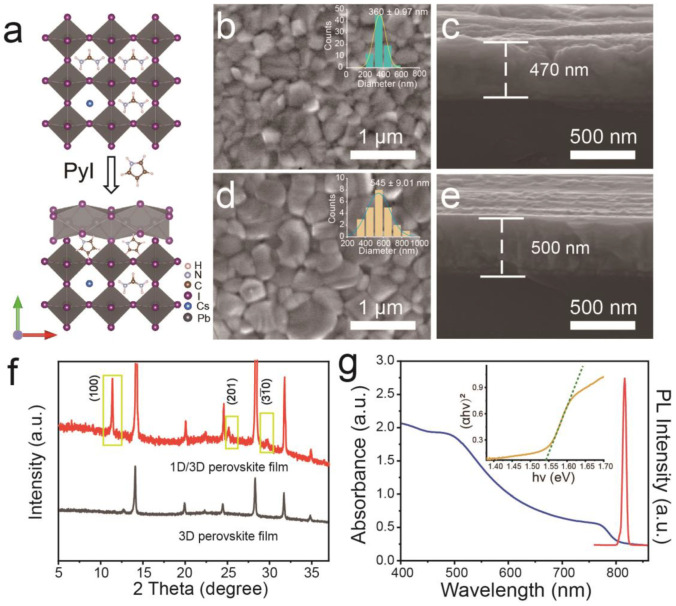
(**a**) Schematic diagram of 1D/3D PyPbI_3_/FACs crystal structure. Top-view (**b**) and cross-sectional (**c**) SEM images of 3D FACs perovskite film. The inset is the corresponding statistical diagram of grain size. Top-view (**d**) and cross-sectional (**e**) SEM images of 1D/3D PyPbI_3_/FACs perovskite film are shown. The inset in (**d**) is the statistical diagram of grains size for top-viewed PyPbI_3_/FACs. (**f**) XRD patterns of 3D and 1D/3D perovskite. (**g**) UV-vis and PL spectra and (inset) corresponding Tauc plot of 1D/3D PyPbI_3_/FACs perovskite film.

Figure 2a depicts the schematic diagram of the 1D/3D hybrid-structured PyPbI_3_/FACs perovskite crystal structure [50]. At the top of the 1D/3D perovskite film, the surface shares a 1D chain of [PbI_3_]^-^ octahedron, and a large number of pyridine cations (Py^+^) are arranged around the chain, which is the crystal structure of PyPbI_3_ [22]. Scanning electron microscopy (SEM) images were performed to reveal the surface morphology effect of 1D PyPbI_3_ on perovskite film. As the top-view SEM image displayed in Figure 2b, 3D FACs perovskite film is composed of densely packed particles with few pinholes but presents a rough surface. Simultaneously, these bare grains present a mass of different-oriented planes. Moreover, the distribution of crystallite size is relatively uniform, with an average value of 360 nm (see the inset in Figure 2b). The thickness of the 3D FACs perovskite layer is about 470 nm, as is shown in the cross-sectional SEM image (Figure 2c). However, after coating on the 1D PyPbI_3_ layer, the crystal particles on 1D/3D PyPbI_3_/FACs perovskite film significantly enlarge, and the corresponding statistical diagram (inset in Figure 2d) shows the crystallite size is about 545 nm. According to the previous reports [45,48], the N-H in Py^+^ cation ions can bind with I in [PbI_3_]^−^ octahedra cage via hydrogen bonding. Thus, the formed PyPbI_3_ is coated on the top of different-oriented planes of FACs grain, which seems to prompt the grain growth. Moreover, the grain size is heterogeneous, and adjacent grains become loose with vast grain boundaries, availing the penetration and contact of gas molecules. The cross-sectional SEM image verifies that the thickness of the 1D/3D hybrid-structured PyPbI_3_/FACs film is larger than that of 3D FACs (Figure 2e), indicating that the 1D layer successfully covers 3D perovskite film.

X-ray diffraction (XRD) patterns were carried out to confirm the crystal structure of the 1D/3D hybrid-structured and 3D perovskite films. As shown in Figure 2f, compared with 3D perovskite (black curve), the XRD pattern of the 1D/3D hybrid-structured perovskite film (red curve) contains all diffraction peaks of 3D FACs perovskite, including 12.7°, 14.1°, 19.9°, 24.5°, 28.3°, 31.7° and 34.9° [51]. Meanwhile, the extra characteristic peaks at 11.2°, 25.1° and 29.6° are well corresponding to the (100), (201), and (3$\bar{1}$0) planes of 1D PyPbI_3_ [22], which suggests the successful construction of the 1D/3D PyPbI_3_/FACs bilayer perovskite film. It is worth mentioning that the diffraction peak of PbI_2_ at 12.7° appears in 3D FACs but disappears in 1D/3D PyPbI_3_/FACs, which may be due to the PyI precursor solution reacting with the residual PbI_2_ in 3D FACs perovskite to form a PyPbI_3_ coating layer. The peak intensity of 1D PyPbI_3_ is relatively weaker in comparison with that of 3D FACs, which is due to the low content of PyI species. This result is consistent with the thinner thickness of PyPbI_3_ in the 1D/3D bilayer perovskite film. Optical properties are important characteristics of perovskite materials. Ultraviolet and visible spectrophotometry (UV-vis) and photoluminescence (PL) of the 1D/3D hybrid-structured perovskite film were tested. In Figure 2g, the optical absorbance spectrum of 1D/3D PyPbI_3_/FACs perovskite shows an onset of the absorbance at about 800 nm with a corresponding bandgap of 1.546 eV, and the strong emission peak in the PL spectrum appears at about 815 nm. Compared with the 3D FACs film (Figure S1), the 1D/3D PyPbI_3_/FACs bilayer film displays similar adsorption in the whole visible wavelength range, but the PL intensity is slightly weakened, which may be due to the increment of defects introduced by PyPbI_3_. These results also suggest that the 1D PyPbI_3_ layer is successfully coated on the 3D FACs perovskite film with negligible optical properties changes.

### 2.2. Gas-Sensing Performances

All detection gas-sensing performances based on 3D FACs or 1D/3D PyPbI_3_/FACs were carried out in a sealed container, which is characterized by recording the resistance signals on a Keithley 2401 source meter. The response of the 3D FACs and 1D/3D PyPbI_3_/FACs bilayer perovskite exposed to 10 ppm NO_2_ was detected under ambient conditions (temperature 25 ± 2 °C and humidity 50 ± 3%).

**Figure 3:**
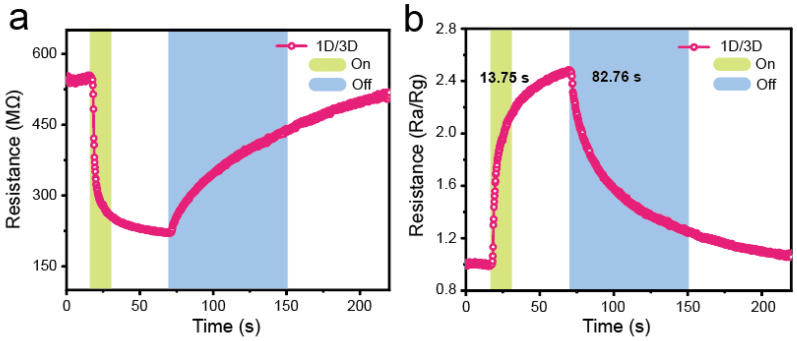
The transient (**a**) resistance and (**b**) response of the 1D/3D PyPbI_3_/FACs perovskite-based sensor toward 10 ppm NO_2_.

Figure 3a and Figure S2a exhibit the transient resistance curve of sensor-based 1D/3D PyPbI_3_/FACs bilayer perovskite and 3D FACs perovskite film toward 10 ppm NO_2_ at a constant applied bias voltage of 5 V. Once exposed to NO_2_ atmosphere (Figure 3a and Figure S2a), the resistance of both the 1D/3D PyPbI_3_/FACs and 3D FACs perovskite-based sensor decrease quickly, and then recovers when NO_2_ is removed, which indicating a reversible interaction between NO_2_ detection gas and perovskite sensor material [29]. In general, the gas response can be obtained by the equation S = R_a_/R_g_, and the corresponding response/recovery time (τ_res_/τ_res_) is defined as the required time to reach 90% of the response value during the response/recovery process [52].

As displayed in Figure 3b, the response and recovery time of the 1D/3D PyPbI_3_/FACs perovskite-based sensor toward 10 ppm NO_2_ is 13.75 s and 82.76 s, respectively, which is slower than that of 3D FACs (seen Supplementary Materials Figure S2b), but the time is faster than most of metal oxide-based sensors [53,54,55,56,57]. A longer recovery time suggests that the desorption process of NO_2_ is relatively difficult, while the response curve at room temperature can automatically return to the initial baseline, indicating the excellent desorption properties of the 1D/3D PyPbI_3_/FACs perovskite-based sensor. Moreover, the 1D/3D PyPbI_3_/FACs perovskite-based sensor to NO_2_ gas displays a stronger response than 3D FACs-based sensors and compared with common metal oxide semiconductor sensors, the operating temperature is room temperature rather than high temperature, which indicates that the 1D/3D PyPbI_3_/FACs perovskite is a promising candidate in the field of low-energy consumption and portable gas sensors.

**Figure 4:**
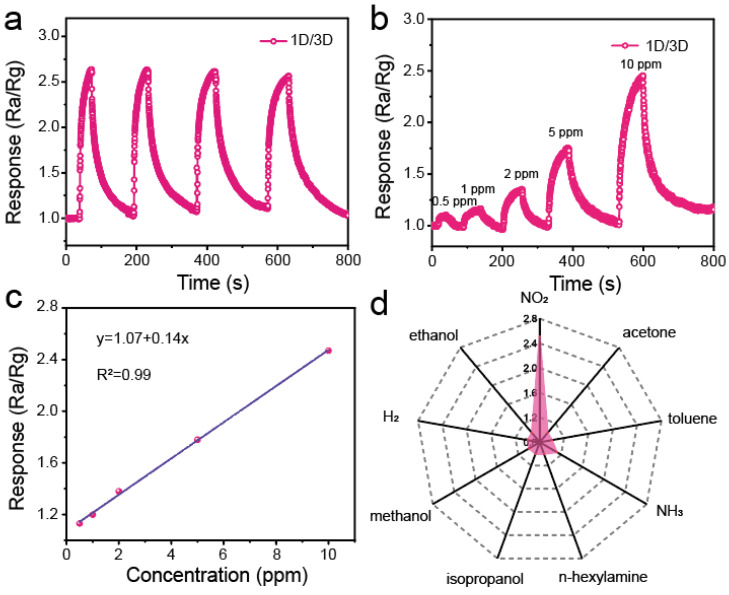
NO_2_-sensing performances of the 1D/3D hybrid-structured PyPbI_3_/FACs perovskite-based sensor: (**a**) reversibility toward 10 ppm NO_2_, (**b**) the transient response toward different concentrations of NO_2_, (**c**) curve of response versus NO_2_ concentrations and (**d**) selectivity to various gases with 10 ppm.

The reversibility and stability of gas sensors are the pivotal parameters that determine their further practical application. Figure 4a and Figure S3 depict the dynamic transient response of sensors based on the 1D/3D PyPbI_3_/FACs and 3D FACs perovskite toward 10 ppm NO_2_ gas at room temperature. After four consecutive cycles, the response of both 1D/3D PyPbI_3_/FACs and 3D FACs perovskite-based sensors toward 10 ppm NO_2_ almost remains at the initial value with negligible attenuation, indicating that the 1D/3D PyPbI_3_/FACs and the perovskite-based sensor has excellent repeatability and reversibility.

The dynamic sensing transients of the 1D/3D PyPbI_3_/FACs perovskite-based sensors toward different concentrations of NO_2_ gas ranging from 0.5 to 10 ppm were manifested in Figure 4b. The transient response of the 1D/3D PyPbI_3_/FACs perovskite-based sensor toward NO_2_ gas rapidly increases with a continuous increase of NO_2_ and then returns to the original baseline state once NO_2_ is removed. The response values of the 1D/3D PyPbI_3_/FACs perovskite-based sensor are 1.13, 1.2, 1.78 and 2.47 toward 0.5, 1, 2, 5 and 10 ppm NO_2_, respectively. The corresponding dynamic resistance variation of the 1D/3D perovskite-based sensor toward different concentrations of NO_2_ is manifested in Figure S4. Similarly, the resistance values of the 1D/3D perovskite-based sensor will decrease when the contact concentration of NO_2_ increases and subsequently recover to baseline after removing NO_2_ gas.

Figure 4c reveals the fitted sensor response of the 1D/3D hybrid-structured PyPbI_3_/FACs perovskite as a function of NO_2_ gas. As shown in Figure 4c, the 1D/3D PyPbI_3_/FACs perovskite-based sensor depicts good linearity in response to low-concentration NO_2_ gas, with a corresponding coefficient of 0.99. The fitted equation derived from the response/recovery curve in Figure 4b is y = 1.07 + 0.14x, which can deduce that the average sensitivity is 0.14 ppm^−1^. The limit of detection (LOD) is defined by the lowest detectable concentration, according to the equation: LOD = 3 × rms/S, wherein rms refers to the root-mean-squared noise of the sensor, and S refers to sensitivity. The calculated LOD of the NO_2_ gas sensor based on the 1D/3D hybrid-structured PyPbI_3_/FACs perovskite can be as low as 220 ppb, suggesting its high sensitivity.

The selectivity of a sensor device is another critical factor owing to the complicated gas compositions in the atmosphere. To prove the selectivity of the device, the response of 1D/3D PyPbI_3_/FACs perovskite sensors to various gases with 10 ppm, including NH_3_, methanol, triethylamine, n-butanol, H_2_, ethanol, acetone and formaldehyde, are investigated under the same environmental conditions. In Figure S5, except for NO_2_, and NH_3_, the resistances of the 1D/3D PyPbI_3_/FACs perovskite-based sensor toward other gases depict no signals. In Figure 4d, the 1D/3D hybrid-structured PyPbI_3_/FACs perovskite-based sensor presents the most remarkable response toward NO_2_. Although the 1D/3D PyPbI_3_/FACs perovskite-based sensor to NH_3_ presents a slight response of 1.12, it is easy to distinguish NH_3_ and NO_2_. It is due to the transient resistance changes of the 1D/3D PyPbI_3_/FACs perovskite-based sensor toward NO_2_ and NH_3_ that it showed the opposite response (Figure 3a and Figure S6) when gas was injected or removed. These results suggest that the 1D/3D PyPbI_3_/FACs perovskite-based sensor shows a superior selectivity toward NO_2_ gas.

**Figure 5:**
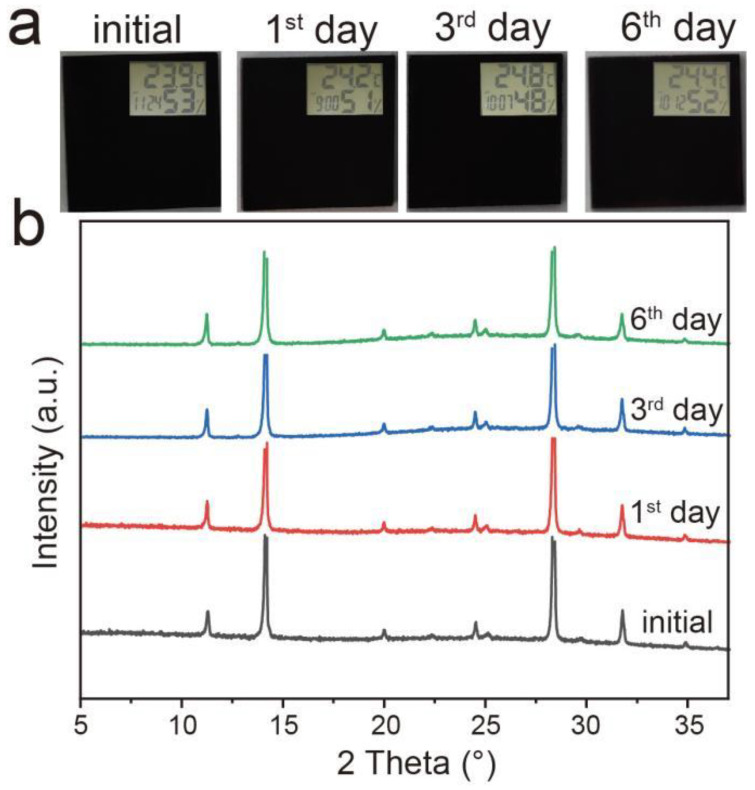
Time-dependent photographs (**a**) and XRD patterns (**b**) of 1D/3D PyPbI_3_/FACs perovskite film. Insets in (**a**) show the measured surroundings, including temperature and humidity.

The long-term stability of a gas sensor is another important factor in determining a large-scale commercial application. Previous perovskite devices are usually stored in a glove box or sealed. Here, to reveal the stability, sensor-based 1D/3D PyPbI_3_/FACs hybrid-structured perovskite material is stored without any encapsulation under high humidity conditions (50 ± 3%). As the photographs manifest in Figure 5a, the 1D/3D PyPbI_3_/FACs hybrid-structured perovskite film still maintains the original macroscopic characteristics well in the successive six days, and no obvious degradation occurs on the surface. The structure and phase changes of the 1D/3D PyPbI_3_/FACs perovskite film are traced by an XRD test. In Figure 5b, no new diffraction peaks appear, and the position and strength of the characteristic peaks in XRD patterns almost remain the same for the consecutive days, but a 3D FACs film without 1D PyPbI_3_ coating layer presents decomposition after three days (Figure S7). These results confirm that 1D PyPbI_3_ coating film significantly enhances the phase stability of 3D FACs perovskite, which may be due to the fact that PyPbI_3_ with a one-dimensional structure as a barrier layer can effectively restrain the intrusion of water molecules into the vulnerable FACs perovskite film to improve the phase stability.

**Figure 6:**
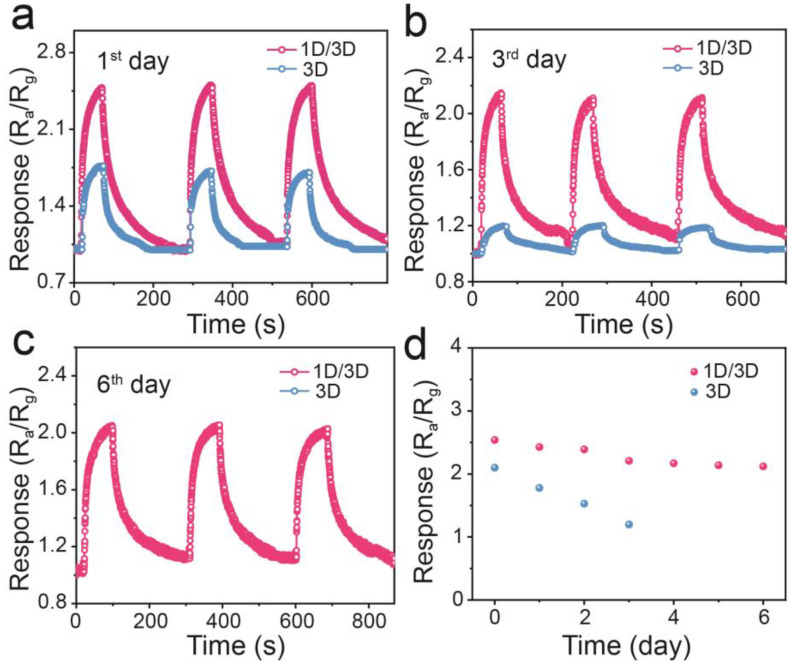
Long-term stability of 1D/3D hybrid-structured PyPbI_3_/FACs and 3D FACs perovskite-based sensor toward 10 ppm NO_2_ recorded at the 1st (**a**), 3rd (**b**) and 6th day (**c**). (**d**) Time-dependent response value.

In addition, gas-sensing tests in Figure 6 reveal the response of the 1D/3D PyPbI_3_/FACs and 3D FACs perovskite-based sensor to 10 ppm NO_2_ gas in the consecutive six days, and the sensor device without encapsulation is kept at room temperature in the ambient environment (temperature 25 ± 2 °C and relative humidity 50 ± 3%). As the transient response curves depicted in Figure 6a–c, the 1D/3D PyPbI_3_/FACs perovskite-based sensor toward 10 ppm NO_2_ still maintains a superior sensing performance with fast response/recovery time and reversibility after being successively exposed to high humidity conditions (50 ± 3%) for 6 days. The initial 3D FACs perovskite-based sensor shows good reversibility (Figure S3), but the 3D FACs perovskite-based sensor exhibits poor stability (Figure 6). The time-dependent response of the 3D FACs perovskite-based sensor toward 10 ppm NO_2_ shows a sharp attenuation. On the 3rd day, the response of the 3D FACs perovskite-based sensor decreased from 2.10 to 1.20 (Figure 6b), suggesting that a sensor device based on 3D FACs perovskite scarcely works. An obvious comparison of the time-dependent stability of 3D FACs and 1D/3D PyPbI_3_/FACs is depicted in Figure 6d, suggesting the superior stability of the 1D/3D PyPbI_3_/FACs perovskite-based sensor. In addition, the responding response of the 1D/3D PyPbI_3_/FACs bilayer perovskite-based sensor is 2.54, 2.21, and 2.12 on the 1st, 3rd and 6th day, respectively, presenting a slight reduction in the successive six days. Although the 1D PyPbI_3_ layer can block the water molecules infiltrating into 3D FACs perovskite, the water molecules in high-humidity conditions inevitably absorb and occupy active sites, which decreases adsorption sites for NO_2_ along with a decline of the response value. More interestingly, the 1D/3D PyPbI_3_/FACs bilayer perovskite-based sensor also displays good stability in a higher humidity environment. The 1D/3D PyPbI_3_/FACs sensor is placed in a higher humidity environment (relative humidity 70 ± 1%) for six successive days. As shown in Figure S8, the 1D/3D PyPbI_3_/FACs perovskite-based sensor in relative humidity of 70 ± 1% displays a similar response tendency to that in 50 ± 3%. The time-dependent response in Figure S8 manifests a good stability of the 1D/3D PyPbI_3_/FACs perovskite-based sensor in a higher humidity condition. The response value in RH of 70 ± 1% is slightly inferior to that in RH of 50 ± 3%, which can ascribe to the decreased sites for NO_2_ due to more water molecules in higher humidity adsorbed on the sites of the perovskite material. Fortunately, the response attenuation of the gas sensor based on a 1D/3D PyPbI_3_/FACs bilayer perovskite can be effectively suppressed by device encapsulation or prepositive dehydration equipment. Thus, the 1D/3D PyPbI_3_/FACs hybrid-structured perovskite-based sensor exhibits a promising application toward low-concentration NO_2_ detection at room temperature.

## 3. Materials and Methods

### 3.1. Chemicals

Cesium iodide (CsI, 99.9999%) was purchased from Aladdin Bio-Chem Technology Co. LTD, Shanghai, China. Lead iodide (PbI_2_) was purchased from Xi’an Polymer, Xi’an, Liaoning, China. Formamidinium iodide (FAI, 99.9%) was purchased from Advanced Election Technology, China. Dimethyl sulfoxide (DMSO) and N,N-dimethylformamide (DMF) were purchased from Sigma-Aldrich, St. Louis, MO, USA. Pyrrolidinium hydroiodide (PyI) was purchased from TCI, Tokyo, Japan. Isopropyl alcohol (IPA, C_3_H_8_O, AR), ethyl acetate (C4H_8_O_2_, AR), methanol, triethylamine, formaldehyde, n-butanol, acetone and ethanol were purchased from Sinopharm Chemical Reagent Co., Ltd, Shanghai, China. All the commercial materials were used as received without further purification.

### 3.2. Fabrication Process of the 1D/3D Perovskite-Based Sensor

An interdigital electrode (electrode spacing: 100 μm, size: 10 × 10 mm^2^, substrate: glass) was prepared by a thermal evaporation method. The detailed procedure is as the following: Firstly, the glass substrate was successively washed with distilled water, ethanol, acetone and 2-propanol for 20 min. Then, clean N_2_ gas was used to blow away the residual liquid. The glass substrate was treated by Ar plasma with a power of 250 W for 5 min. After that, Au as an evaporated source is deposited on the top of a clean glass substrate under a pressure of 5 × 10^−5^ Pa in a vacuum chamber to obtain an interdigital electrode. 

The 1D/3D perovskite film on the interdigital electrode was prepared via a two-step spin-coating process. Firstly, for the mixed A cation FA_0.83_Cs_0.17_PbI_3_ (abbreviated as FACs in the main text) metal halide perovskite layer, a one-step spin-coating method was adopted as previously reported [51]. In detail, to prepare FACs film, the precursor solution was first prepared by dissolving 1 mmol FAI (170 mg), 0.2 mmol CsI (56 mg) and 1.2 mmol PbI_2_ (552 mg) in 1 mL mixed solvent containing DMF (700 μL) and DMSO (300 μL). The prepared solution was stirred for 2 h at room temperature to obtain a clear and transparent liquid. Before the preparation of the FACs thin film, the precursor solution was filtered through a 0.22 µm poly tetra fluoroethylene (PTFE) filter. Then, 50 μL precursor solutions were spin-coated at 1000 rpm for 10 s and then at 6000 rpm for 30 s via a one-step method. Additionally, 110 μL of EA was dripped on the film to extract the solvent at 12th s prior to the end of the process. Then, the film was annealed at 170 °C for 10 min to obtain a 3D FACs film.

For the 1D/3D PyPbI_3_/FACs hybrid-structured perovskite film, 50 μL of PyI (10 mg PyI in 1 mL IPA) solutions were spin-coated on top of the FACs perovskite layer at 3000 rpm for 30 s, then the film was annealed at 120 °C for 10 min. 

The whole process was carried out under ambient conditions, and the devices were stored under ambient conditions (temperature 25 ± 2 °C; humidity 50 ± 3%) without any encapsulations.

### 3.3. Characterization

The surface morphology of 3D FACs and 1D/3D PyPbI_3_/FACs perovskite was characterized by using scanning electron microscopy (SEM, Regulus-8100, HITACHI, Tokyo, Japan) operating at 20 kV. The crystal structure was investigated by X-ray diffraction (XRD, DX2700, Dandong Kemait NDT C., Ltd, Liaoning, China) with a 2θ ranging from 5 to 37° in a step of 5° min^−1^, using a monochromatic Cu Kα radiation source. Before the 3D FACs and 1D/3D PyPbI_3_/FACs perovskite were exposed to gases, the XRD measurement was held on a glass substrate different from the deposited material on electrodes in order to avoid any interaction of sensing elements from the X-ray beam. The absorbance spectrum of the 3D FACs and PyPbI_3_/FACs perovskite were measured by UV-visible spectroscopy (T9, PERSEE, Beijing, China) in the wavelength ranging from 400 to 870 nm on a quartz glass substrate. The photoluminescence (PL) spectrum of 3D FACs and 1D/3D PyPbI_3_/FACs hybrid-structured perovskite were investigated with a Hitachi F-4600 spectrofluorometer, Tokyo, Japan.

### 3.4. Gas-Sensing Test Parameters

The gas-sensing test was collected by a Keithley 2401 source meter. The environmental test conditions were as follows: the pressure was 101,325 Pa, the working temperature was about 25 ± 2 °C and the relative humidity was about 50 ± 3%. A sealed chamber with a volume of 18 L is used for gas sensor testing, and an air inlet and outlet are located in the chamber. Dry NH_3_, H_2_ and NO_2_ were supplied by a sealed gas bag. After injecting the detection gas into the closed box, a built-in fan was used to diffuse the detection gas. When the target source is gas, the volume of injected gas is calculated by the formula

Vg = cV_0_/c_0_

where c is the measured concentration in the test chamber, c_0_ is the concentration of the target gas and V is the volume of the sealed chamber (18 L).

While the target source was liquid, such as methanol, ethanol, n-butanol, acetone, triethylamine and formaldehyde, the detection gases were obtained by evaporating the corresponding injected pure liquids. The required amount of target liquid was dropped onto the micro-heating plate via a microsyringe, and the required liquid volume was calculated by the formula

V_L_ = PV_0_cM/ρRT

where P is the standard atmospheric pressure (P = 101.325 kPa), V_0_ is the volume of the sealed chamber (18 L), c is the gas concentration, M is the molar mass, R is the gas constant (R = 8.314 J/(mol·K)), T the ambient temperature and ρ is the density of the liquid.

## 4. Conclusions

In this work, a 1D/3D PyPbI_3_/FACs bilayer perovskite-based sensor is constructed by post-spin-coating a PyI precursor on the top of a 3D FA_0.83_Cs_0.17_PbI_3_ perovskite film under an ambient atmosphere. The introduced 1D PyPbI_3_ coating layer can not only stabilize the 3D FACs perovskite phase but also produce loose grain boundaries to avail the penetration and contact of gas molecules. Consequently, the 1D/3D PyPbI_3_/FACs bilayer perovskite-based sensor shows a superior selectivity and sensitivity to NO_2_ at room temperature, with a low detection limit of 220 ppb. Moreover, compared with the 3D FACs-based sensor, the response and stability of the 1D/3D PyPbI_3_/FACs hybrid-structured perovskite-based sensor have been significantly improved. Exposed in a high humidity environment (50 ± 3%) for six consecutive days, the 1D/3D PyPbI_3_/FACs perovskite-based sensor toward 10 ppm NO_2_ can rapidly respond with a slight attenuation. This work opens up a new approach to designing and constructing high-stability sensors to detect NO_2_ at room temperature, accelerating its further practical application.

## Data Availability

All data are available upon reasonable request from the corresponding author.

## Supplementary Materials

The following supporting information can be downloaded at: https://www.mdpi.com/article/10.3390/molecules28062615/s1, Figure S1: (a) UV and (b) PL images of 3D FACs and 1D/3D PyPbI3/FACs hybrid-structured perovskite film. Figure S2: The transient (a) resistance and (b) response of the 3D FACs perovskite-based sensor toward 10 ppm NO2. Figure S3: The reversibility of 3D FACs perovskite-based sensor toward 10 ppm NO2. Figure S4: The dynamic resistance variation of the 1D/3D PyPbI3/FACs hybrid-structured perovskite-based sensor towards different concentrations of NO2 gas. Figure S5: The response of PyPbI3/FACs hybrid-structured perovskite sensor toward different gases with 10 ppm. Figure S6: The transient resistance of 1D/3D PyPbI3/FACs hybrid-structured perovskite-based sensor toward 10 ppm NH3. Figure S7: Photograph of 3D FACs film recorded on the 3rd day after exposure in high humidity conditions; Figure S8: Long-term stability of 1D/3D hybrid-structured PyPbI3/FACs and 3D FACs perovskite-based sensor toward 10 ppm NO2 recorded on the 1st (a), 3rd (b) and 6th day (c). (d) The time-dependent response values for 6 successive days in an RH of 70 ± 1% conditions.

## Sample Availability

Samples of the compounds are available from the authors.
