# Supplementary material for: Hybrid 1D/3D-Structured Perovskite as a Highly Selective and Stable Sensor for NO2 Detection at Room Temperature
Source: Molecules. 2023 Mar 13;28(6):2615. doi: 10.3390/molecules28062615 (PMC10051054; doi:10.3390/molecules28062615)
Supplement: Supplementary file 1 [file molecules-28-02615-s001.zip › molecules-2258076-supplementary.pdf]

Supplementary Materials

Hybrid 1D/3D-Structured Perovskite as a Highly Selective and Stable Sensor for NO_2 Detection at Room Temperature

Anqi Cheng, Jinru Zhao, Xi-Ao Wang, Zhen Lu, Yan Qi * and Jiankun Sun *

College of Chemistry and Chemical Engineering, Qingdao University, Qingdao 266071, China;

* Correspondence: qddxqy@qdu.edu.cn (Y.Q.); sunjk@qdu.edu.cn (J.S.)

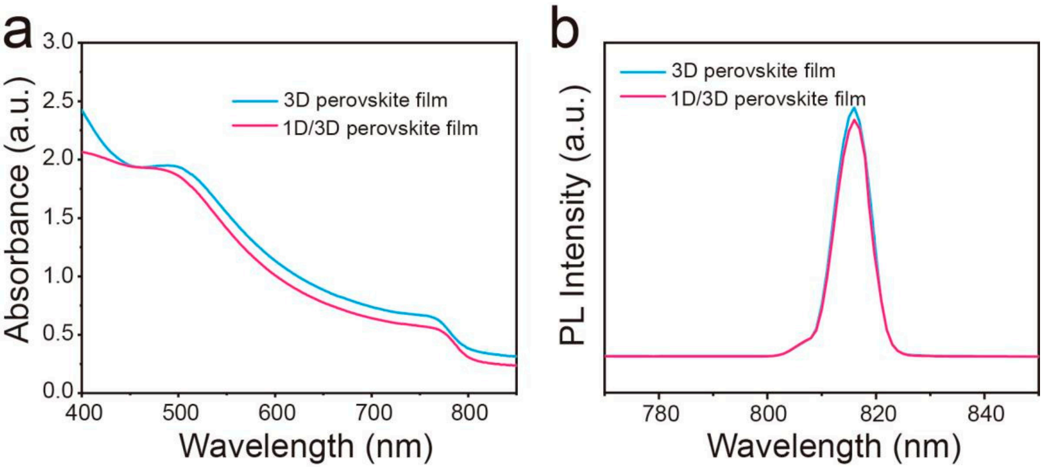

Figure S1. (a) UV and (b) PL images of 3D FACs and 1D/3D $PyPbI_3$/FACs hybrid-structured perovskite film.

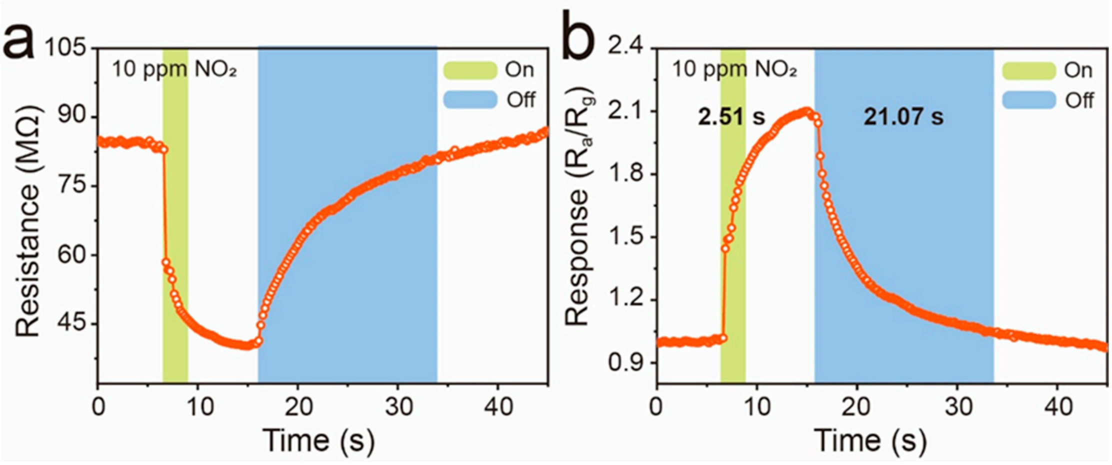

Figure S2. The transient (a) resistance and (b) response of the 3D FACs perovskite-based sensor toward 10 ppm NO_2.

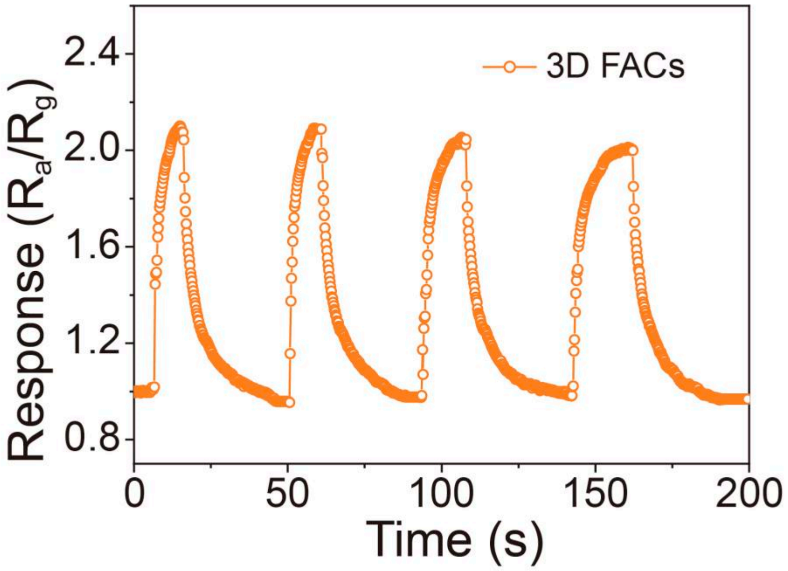

Figure S3. The reversibility of 3D FACs perovskite-based sensor toward 10 ppm NO_2.

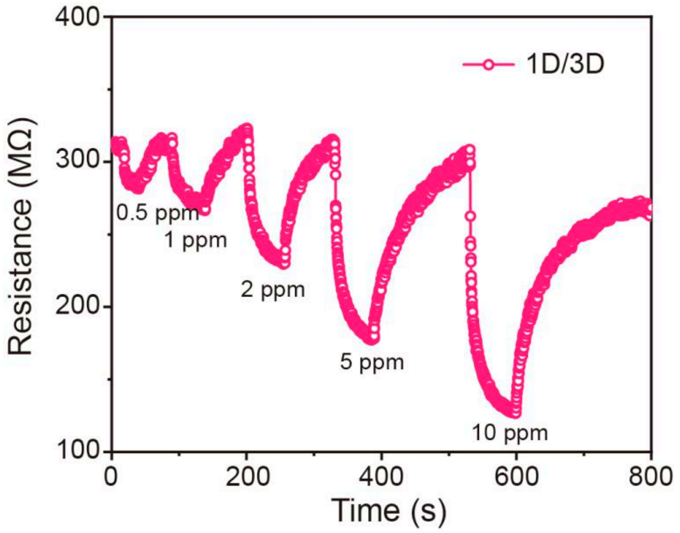

Figure S4. The dynamic resistance variation of the 1D/3D $PyPbI_3$/FACs hybrid-structured perovskite-based sensor towards different concentrations of NO_2 gas.

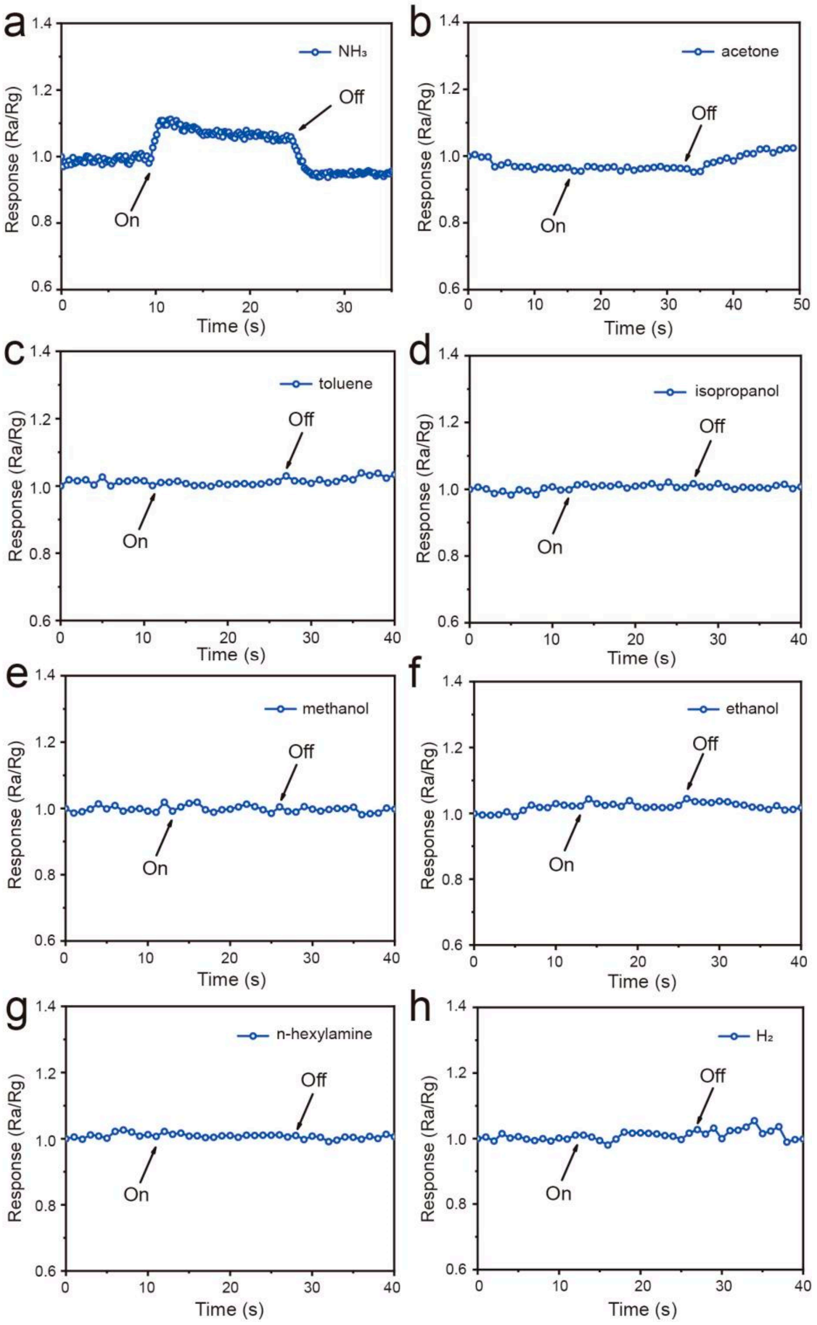

Figure S5. The response of $PyPbI_3$/FACs hybrid-structured perovskite sensor toward different gases with 10 ppm (a–h).

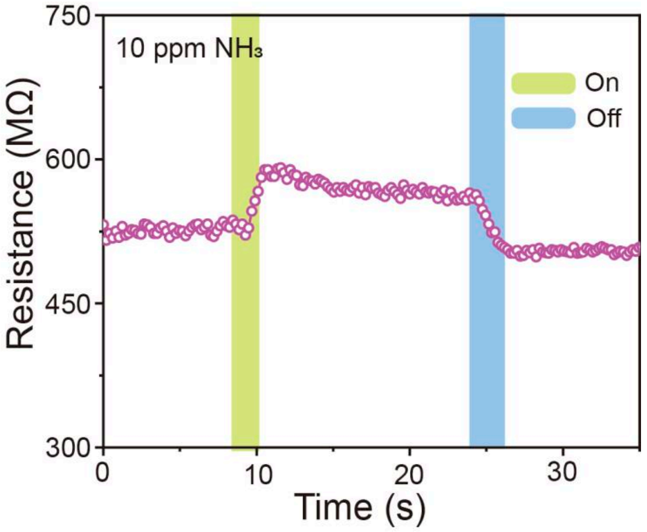

Figure S6. The transient resistance of 1D/3D $PyPbI_3$/FACs hybrid-structured perovskite-based sensor toward 10 ppm NH_3.

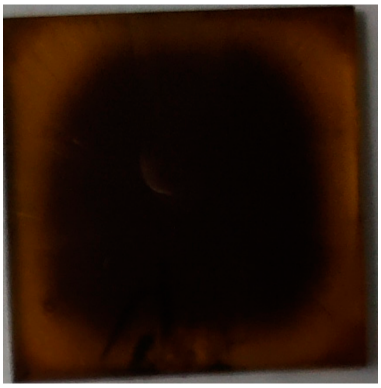

Figure S7. Photograph of 3D FACs film record at 3rd day after exposure in high humidity conditions.

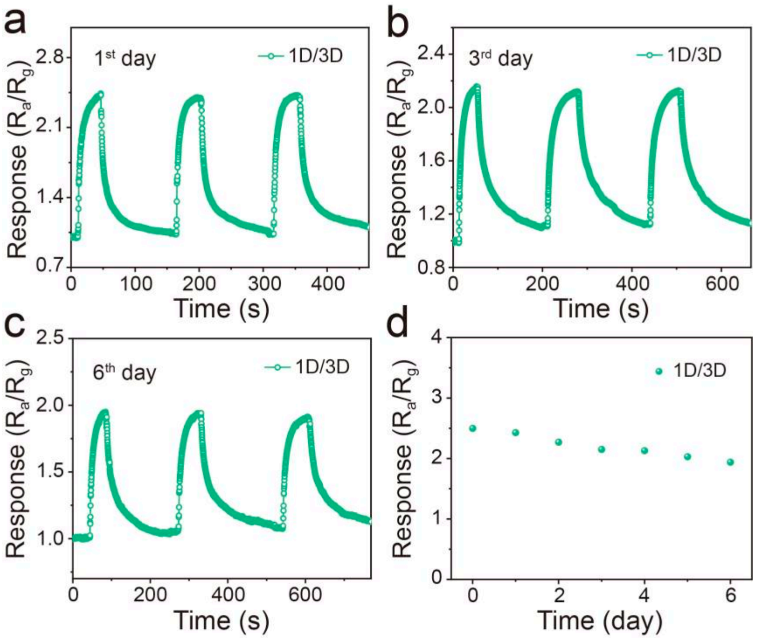

Figure S8. Long-term stability of 1D/3D hybrid-structured $PyPbI_3$/FACs and 3D FACs perovskite-based sensor toward 10 ppm NO_2 recorded at the 1st (a), 3rd (b) and 6th day (c). (d) The time-dependent response values for 6 successive days in an RH of 70 ± 1% conditions.
